# Social determinants of health in pregnant individuals from underrepresented, understudied, and underreported populations in the United States

**DOI:** 10.1186/s12939-023-01963-x

**Published:** 2023-09-06

**Authors:** Guillermina Girardi, Monica Longo, Andrew A. Bremer

**Affiliations:** 1 Pregnancy and Perinatology Branch, Eunice Kennedy Shriver National Institute of Child Health and Human Development, Bethesda, MD USA; 2 Pediatric Growth and Nutrition Branch, Eunice Kennedy Shriver National Institute of Child Health and Human Development, Bethesda, USA

**Keywords:** Social determinants of health, Health inequities, U3 populations, Stress, Pregnancy, Neonatal outcomes

## Abstract

Women represent the cornerstone of a family’s overall health. Therefore, supporting women’s health, particularly in pregnancy, is important to promote public health. Emerging data highlight the contribution of social determinants of health (SDOH) on pregnancy outcomes in understudied, underrepresented, and underreported (U3) populations. Importantly, women are uniquely affected by and more vulnerable to adverse outcomes associated with SDOH. The maternal mortality rate has also increased significantly in the United States, especially among U3 individuals. Factors such as access to safe food, housing and environment, access to education and emergency/health services, and stressors such as interpersonal racism, poverty, unemployment, residential segregation, and domestic violence may make women from U3 populations more vulnerable to adverse reproductive health outcomes. Despite progress in promoting women’s health, eliminating social and health disparities in pregnant individuals remains an elusive goal in U3 populations. Moreover, chronic exposure to excessive social/cultural stressors may have a physiologic cost leading to pregnancy complications such as miscarriages, preterm birth, and preeclampsia. Thus, the identification of SDOH-related factors that drive differences in pregnancy-related complications and deaths and the implementation of prevention strategies to address them could reduce disparities in pregnancy-related mortality in U3 populations.

Women represent the cornerstone of a family’s overall health. While research shows that investing in women’s health results in a healthier overall population, healthier future generations, and greater social and economic benefits [1], maternal mortality remains unacceptably high in the United States (U.S.) [2, 3]. According to a recent report from the U.S. Centers of Disease Control and Prevention (CDC) [2], the maternal mortality rate increased significantly in 2020 (from 20.1 deaths per 100,000 live births in 2019 to 23.8 in 2020). Women of color in the US, regardless of economic or educational status, are three to five times more likely to die from pregnancy-related causes than white women. The mortality rate increased from 44.0 to 55.3 per 100,000 live births in black women, while among Hispanic women the rate increased from 12.6 to 18.2 per 100,000 from 2019 to 2020 [2].

A recent study based on comprehensive mortality surveillance for all states via vital registration identified significant disparities in different states and racial and ethnic groups [4]. Black, American Indian and Alaska Native pregnant individuals are particularly at increased risk for adverse outcomes [4]. These inequities in the U.S. are also evident in pregnant individuals of economically disadvantaged groups, women who live in underserved rural populations, and women of sexual and gender minority groups.

Despite the need for research to understand and reduce these inequities, women of these populations remain largely Understudied, Underrepresented, and Underreported in biomedical research. The Office of Research on Women’s Health (ORWH), established in 1990 and the first Public Health Service office dedicated specifically to promoting women’s health research within and beyond the National Institutes’ of Health (NIH) scientific community, developed the U3 (Understudied, Underrepresented and Underreported) framework to draw attention to the lack of research on persistent disparities in women’s health and healthcare and to support research to address this gap [5].

One potential mediator of the disparities observed in U3 populations is social determinants of health (SDOH), including: *(i)* individual socioeconomic factors; *(ii)* community factors such as crime, poverty, housing, and the racial/ethnic makeup of the community; and *(iii)* the physical environment. Factors such as access to education and emergency/health services and stressors such as interpersonal racism, poverty, unemployment, residential segregation and domestic violence may also increase the vulnerability of women from U3 populations to adverse reproductive health outcomes. Moreover, chronic exposure to excessive social/cultural stressors can have a physiologic cost leading to pregnancy complications such as miscarriages, preterm birth, and preeclampsia.

Despite progress in promoting women’s health, eliminating social and health disparities in pregnant individuals remains an elusive goal in U3 populations. In order to develop potential interventions to reduce these disparities and improve pregnancy outcomes, it is important to understand the underlying mediators, or causes, for these observed differences. Herein we will discuss SDOH as mediating factors in health inequities in women from U3 groups and how ensuring the implementation of support programs and research may help achieve health equity in pregnant individuals.

## Biological, social, and cultural factors responsible for health inequities in pregnant individuals

### Biological factors

Genetics variations may contribute to increased susceptibility to certain adverse pregnancy outcomes such as preterm birth and preeclampsia in some ethnic groups [6–9]. For example, genetic polymorphisms have been associated with preeclampsia and preterm deliveries in African-American women, and American Indian or Alaskan Native women are also widely reported to be disproportionately affected by preeclampsia [6, 7]. However, while ethnicity-related genetic factors may a play a role in the susceptibility of some individuals from U3 populations to adverse pregnancy outcomes, a combination of biological, social, and cultural factors, and not race alone, is likely to contribute to the higher rates of pregnancy complications in U3 populations.

### Social determinants of health

Emerging data highlight the contribution of SDOH on a wide range of health risks including the risk for adverse pregnancy outcomes. The World Health Organization (WHO), as well the CDC, defines SDOH as the conditions in which people are born, live, learn, work, and age, and the wider set of forces and systems shaping the conditions of daily life [10, 11]. SDOH are important contributors to health disparities and inequities and may also have serious implications on health outcomes for pregnant individuals. Specifically, SDOH have a major impact on women’s health, well-being, and quality of life. SDOH can also affect women’s health at different levels, at individual/patient level and also at community level. Among the SDOH factors with significant effects at the individual level, socioeconomic status (income, marital status, access to health insurance and quality health care, access to healthy food) and education, housing insecurity, the physical environment, and experience with racism/racial discrimination are very prevalent. SDOH that contribute to women’s health inequities with impact at the community level include: poverty level, crime, exposure to polluted air and water, language barriers and low literacy skills [11]. SDOH associated with institutionalized and interpersonal racism, including poverty and unemployment, may make women from U3 populations more vulnerable to adverse reproductive health outcomes [11]. Furthermore, residential segregation and discriminatory practices such as “redlining” are associated with lower-quality schools and health care and can also affect the wellbeing of U3 pregnant individuals and their offspring [12].

Importantly, women are uniquely affected by and more vulnerable to adverse outcomes associated with SDOH. For example, women are paid less than men for comparable employment and this gap is wider for Latina, Native American, and Black women [13]. With lower incomes to support their families, women from U3 populations need to make important decisions and are often forced to choose between essential needs such as housing, childcare, food, and health care. Socioeconomic factors are also important stressors that might affect pregnant individuals. For example, a nationwide study of over 1 million births reported large inequalities in pregnancy outcomes between ethnic and socioeconomic groups in England, despite the fact that all women received comparable medical care at the English National Health Service (NHS), a publicly funded healthcare system [14]. This study is particularly important in indicating that factors other than health care access and quality, such as a woman’s educational, socioeconomic, and ethnic background, might be related to serious adverse pregnancy outcomes. The authors also suggested the existence of a cumulative impact of racism and social and economic inequalities on the health of pregnant people [14]. In the U.S., these factors might be amplified by the inability of women from U3 populations to afford health insurance. Mainly due to financial barriers, racial–ethnic minority women experience higher rates of uninsurance than white non-Hispanic women, from preconception to postpartum [15]. Differential insurance coverage may have important implications for racial–ethnic disparities in access to perinatal care and maternal–infant health. Among the sociodemographic and preconception/prenatal health factors that drive disparities in preterm birth among women of color, the largest contributors included maternal education and marital status/paternity acknowledgment [16].

Stress may be defined as environmental demands that exceed the adaptive capacity of an organism, resulting in biological and psychological changes that may have detrimental effects on health. Stressors in U3 communities can take many forms, including those associated with economic difficulties, physical deprivation, low social status, occupational strain, neighborhood instability, and discrimination. Research has shown that poor maternal and infant health outcomes result from chronic exposure to these stressors, including higher rates of perinatal depression and preterm birth [17]. Stress also generates biological responses leading to suppression of reproductive functions [18]. Specifically, social stressors may affect reproductive health in women by affecting stress hormones (epinephrine, norepinephrine, and dopamine) at the brain level, the hypothalamic-pituitary-adrenal (HPA) axis, and the hypothalamic-pituitary-gonadal (HPG) axis, all of which may lead to altered levels of cortisol, sex steroids, and myriad other hormones. Moreover, these endocrine changes may contribute to hyperglycemia, hypercholesterolemia, and hypertension, all of which have been associated with adverse reproductive outcomes [19–21].

In addition, depressive symptoms resulting from chronic exposure to stressors during pregnancy have been associated with a number of adverse pregnancy outcomes, including preterm birth, hypertension, Cesarean delivery, low birth weight, and neonatal intensive care unit (NICU) admission, all of which have social and emotional impacts on the infants [22]. As currently exemplified by the war in Ukraine, pregnant individuals fleeing violence face innumerable challenges given their unique health status. Specifically, pregnant refugees are uniquely vulnerable to higher rates of preterm birth, preeclampsia, and stillbirth [23]. In addition to the adverse pregnancy and neonatal outcomes, mental health problems for the mothers may also persist after birth.

Structural racism may also negatively impact pregnancy and neonatal outcomes. For women of color, structural inequality, lack of opportunities, discrimination, and systemic racism have been shown to be associated with increased mortality during pregnancy and childbirth [24–26]. Furthermore, a recent study found that women of color perceive their interactions with doctors, nurses, and midwives as being misleading, limiting their maternity health care choices [27]. A previous study from this same group of investigators also found that pregnant people of color at risk for preterm birth described being the target of disrespect, racism, and discrimination during healthcare encounters [28]. In addition, recent reports in mainstream media have described the perceptions of pregnant people from U3 populations of being ignored and devaluated in the reproductive healthcare system [29]. These feelings may affect well-being, health behaviors, and the desire to engage and follow clinical recommendations of health care practitioners, ultimately affecting pregnancy outcomes. Therefore, racism-related stresses experienced by women from U3 populations may increase the risk for adverse pregnancy outcomes, such as premature births and hypertensive disorders of pregnancy.

### Cultural factors

Different cultures have different values, beliefs, and practices, and cultural background might compromise reproductive health. For example, many pregnant individuals believe it’s important to follow the traditional pregnancy and birth practices of their culture (such as avoiding certain foods and performing little activity/exercise), even if it’s not what is recommended to them by their healthcare provider.

Patriarchal cultures may also create gender norms that prevent women from making their own decisions and therefore neglect their health [30]. For example, some women are not allowed to seek health care without their husbands’ or other family members consent. Cultural stigma in certain communities is also a contributing factor to adverse maternal outcomes. In certain communities, mainly rural, husbands insist on their women doing difficult household chores when they are pregnant [30]. Marital status is another factor that might contribute to health inequity in pregnant people. 41% of mothers are the sole or primary economical support for their families [31]. Even more, among U3 populations, a large percentage of mothers are single mothers. In 2020, there were about 4.25 million Black families in the U.S. with a single mother [31]. Due to lower wages and the absence of a partner, women of U3 populations are also more likely to lack adequate childcare and have limited transportation options that may affect attendance to doctor’s appointments.

Paternal involvement, recognized to have a positive impact on pregnancy and infant outcomes, varies among different cultures. The presence of supporting fathers during pregnancy has been associated with diminished maternal negative health behaviors and risk of preterm birth, low birth weight, and significant reduction in fetal growth restriction [32–34]. Paternal involvement has also been associated with diminished infant mortality up to one year after birth [35]. While father’s involvement in prenatal care is associated with health benefits for both mothers and children, low paternal involvement in prenatal care remains a challenge, in particular among men of color. Recent reports have highlighted the role of the father in pregnancy outcomes in African Americans [36]. Specifically, black women with absent fathers had the highest risk of low birth weight, very low birth weight, preterm birth, very preterm birth, and an infant born small for gestational age (SGA) [37]. Therefore, promoting paternal involvement during the perinatal period may provide a means to decrease the proportion of infants born with a very low birth weight or very preterm, thus potentially reducing the black-white disparity in infant mortality [38].

Women disproportionately experience intimate partner violence, and this is exacerbated in U3 populations from patriarchal cutlures [39]. The patriarchal cultural beliefs and traditions that emphasize male assertiveness and domination of women are frequently associated with domestic violence. Domestic violence is thus another source of stress that might account for adverse pregnancy outcomes and even the death of pregnant individuals. To date, more than half of adult female of color homicides are related to intimate partner violence, and the presence of firearms within those relationships is a key risk factor for intimate partner homicide [40]. Pregnant people in the U.S. are twice as likely to die by homicide than pregnancy-related causes and the majority of these homicides are carried out with firearms. In addition, pregnant and postpartum Black individuals are at the greatest risk [41]. Women from U3 populations also frequently express a high need for support due to intimate partner violence. However, previous experiences of racism might cause women from U3 populations not to trust law-enforcement agencies, thereby preventing them from reporting domestic violence and increasing the risk of adverse pregnancy outcomes and homicide. Moreover, it has been reported that residence in neighborhoods with high firearm violence was associated with higher prevalence of preterm birth [42]. It has also been suggested that lack of opportunities and financial barriers in people of color are root causes of domestic violence. Indeed, limited access to economic opportunities, the inability to build intergenerational wealth, inequalities in healthcare and education, and a sense of feeling unsafe from governmental systems and pervasive racist policies may increase the prevalence of risk factors for domestic violence in people of color.

## Interventions and research priorities

Increased risk, lower-quality care, and socioeconomic disadvantages are important factors in the increased vulnerability of U3 people to adverse pregnancy outcomes [43]. But specific interventions and research priorities addressing these factors may remedy the adverse effects of racism, segregation, and inequality. While the contribution of genetics is likely to play a role in differential risk for adverse pregnancy outcomes [6–9], gene–environment interactions may be responsible for the differences. As such, improving and adherence to screening, potential preventive measures, and treatment recommendations may help reduce differences in adverse pregnancy outcomes. Research into: *(i)* interventions to improve access to health care; *(ii)* to identify and treat risk factors and diagnose and treat infections, inflammation, mental health issues, and nutritional deficiencies; and *(iii)* to provide financial and social support for women to reduce stress may all have significant impact.

Quality of health care is also a decisive and potentially modifiable factor contributing to disparities in pregnancy outcomes. Suboptimal health care may result from racism, segregation, and inequality and adversely affect outcomes. For example, U3 pregnant people and their neonates may receive care in different maternity centers and NICUs with lower quality or organizational models or clinical processes that lead to lower-quality care. In addition, the quality of care received by mothers and infants can differ by race and ethnicity in individual health centers. This may result from organizational processes including cultural factors and communication [44].

Interventions to promote father involvement by increasing men’s feelings of comfort and support in prenatal settings may alleviate maternal stress and have positive impacts on pregnancy outcomes. Furthermore, intervention to increase men’s comfort and perceived expectations of involvement in prenatal care settings may increase paternal confidence in their ability to be a good parent and lead to greater intentions to engage in healthy behaviors along with their partner. To prevent abuse during pregnancy, domestic violence screening and referral training programs and materials to health care personnel who may come in contact with individuals during or after pregnancy should be implemented. Importantly, the time during and after a pregnancy represents an important window of opportunity to identify a person who may be experiencing violence and refer them to needed resources and support, particularly for those individuals who might not otherwise be in contact with various social and economic services. In addition, routine assessment for abuse and gun access should be implemented to protect women’s safety and prevent further trauma and potential homicide. Knowing that the severity of abuse to pregnant individuals is highly associated to gun access of the perpetrator [45], initiatives to remove firearms from domestic violence perpetrators may have a positive impact on pregnant people’s health. For example, it has been demonstrated that state laws prohibiting possession of firearms and not allowing the possession of firearms by people convicted of domestic violence-related misdemeanors were associated with substantial reductions in homicide of pregnant and postpartum individuals [46].

Among the social and behavioral factors that influence health and mortality only smoking and alcohol use are commonly assessed by primary care physicians. Only recently have clinicians started to attend to determinants that are often ignored or overlooked in clinical practice. Adoption and meaningful use of electronic health records to obtain and store digitized information on standard measures of social and behavioral determinants and to make the data accessible to clinicians should be encouraged with appropriate attention to privacy and security [47]. Furthermore, a committee of social scientists, clinicians, and informaticians, convened by the Institute of Medicine, identified race, ethnicity, education, financial strain, stress, depression, social connection or isolation, intimate partner violence, and tobacco and alcohol use as the factors most strongly associated with health and assessed the availability and use of standardized measures of those determinants [48].

Research focused on gene–environment interactions and epigenetic mechanisms shows promise in maternal and neonatal health by showcasing the importance of fetal development in socioeconomic disadvantaged populations exposed to excesive stress. Importantly, a better understanding of the deleterious effects of social factors during pregnancy will help the promotion of more effective prevention and intervention strategies to optimize the mother’s and infant’s health. A meaningful connection and collaboration between health care providers and the community is also imperative to protect pregnancies and the health of the offspring.

### Implementation of programs to address health inequities in pregnant individuals

Addressing social determinants of health can lead to reductions in health disparities in pregnant individuals. Identifying the underlying factors of SDOH that contribute to disparities is crucial to achieving health equity and improving maternal and neonatal outcomes. Table 1 shows some key gaps and measures that can be taken to address these issues.

**Table 1:** Factors affecting health equity in pregnant individuals

Gap		Measures to implement
Education	Limited access to quality education	Improve educational opportunities for women by investing in girls’ education, promoting gender equality in schools and colleges, and providing scholarships and mentorship programs.
Economic empowerment	Women often face gender-based discrimination in the workplace, leading to lower pay, limited job opportunities, and financial insecurity.	Implement policies that ensure equal pay for equal work, promote women’s entrepreneurship and leadership, provide affordable childcare facilities, and support vocational training and skill development programs
Healthcare access	Limited access to affordable and quality preconception and prenatal care. Limited acces to mental health services Limited access to treatment for substance	Expand healthcare coverage and reduce financial barriers through policies like universal healthcare, increase the number of healthcare facilities in underserved areas, improve women’s access to reproductive health services, and enhance cultural competency and sensitivity in healthcare provision
Housing access	Housing insecurity Homelessness	Inproving housing quality, stability and affordability Improving neighborhood conditions
Violence Prevention	High rates of violence against women, including domestic violence, sexual assault, and gender-based violence	Strengthen laws and enforcement mechanisms to protect women from violence, raise awareness about gender-based violence and its consequences, provide support services for survivors, and promote community-based prevention programs
Social Support Networks	Inadequate social support networks and community resources for women, particularly those facing multiple vulnerabilities	Develop and strengthen community-based organizations and support networks that address the specific needs of women, including immigrant women, women of color, and marginalized groups. This can include peer support groups, counseling services, and community outreach programs
Policy and Advocacy	Insufficient attention to gender-sensitive policies and lack of representation of women in decision-making processes	Develop and implement policies that address gender inequalities, promote gender mainstreaming in all sectors, ensure equal representation of women in leadership positions, and support women’s participation in policymaking and advocacy efforts
Research and Data	Limited research and data on women’s health issues, particularly those related to social determinants of health	Invest in research that explores the intersectionality of gender, race, and socioeconomic factors in health outcomes, collect gender-disaggregated data, and use evidence-based research to inform policy and program development

## Conclusion

Given our current lack of understanding of how SDOH affect women of reproductive age, it is imperative to aid and support women of reproductive age with SDOH-related needs. Some areas of need that have been identified are food to feed their families, utilities, transportation, employment, childcare, housing, and education [49]. The identification of factors that drive differences in pregnancy-related complications and deaths and the implementation of prevention strategies to address them could reduce disparities in pregnancy-related mortality in U3 populations. Specifically, improving women’s health and access to quality care in the preconception, pregnancy, and postpartum periods should be implemented in all U3 individuals. To effectively mitigate the impact of SDOH on women’s reproductive health, training and educating health care providers to assess and address SDOH-related needs is also pivotal. Frontline reproductive health care providers are important as they are frequently the first to contact and engage with women from U3 populations.

At the National Institutes of Health (NIH), the “Implementing a Maternal Health and PRegnancy Outcomes for Everyone (IMPROVE)” Initiative, launched in 2019, addresses these issues and uses an integrated approach to understand biological, behavioral, sociocultural, and structural factors to support research on how to reduce preventable maternal morbidity, decrease severe maternal morbidity, and promote health equity. Ensuring the implementation of culturally-appropriate programs targeting modifiable SDOH, research, and treatment efforts such as therapy, stress management, or mindfulness interventions may also help in achieving health equity. To close, systemic racism and discrimination, as well as myriad other social issues, need to be addressed in order to improve women’s social circumstances, social support, and health throughout their lives.

## Data Availability

Not applicable.
